# A Fluorescent Activatable AND‐Gate Chemokine CCL2 Enables In Vivo Detection of Metastasis‐Associated Macrophages

**DOI:** 10.1002/anie.201910955

**Published:** 2019-10-11

**Authors:** Antonio Fernandez, Emily J. Thompson, Jeffrey W. Pollard, Takanori Kitamura, Marc Vendrell

**Affiliations:** ^1^ Centre for Inflammation Research The University of Edinburgh 47 Little France Crescent EH16 4TJ Edinburgh UK; ^2^ MRC Centre for Reproductive Health The University of Edinburgh 47 Little France Crescent EH16 4TJ Edinburgh UK

**Keywords:** fluorophores, imaging, immunology, molecular logic, tumour microenvironment

## Abstract

We report the novel chemical design of fluorescent activatable chemokines as highly specific functional probes for imaging subpopulations of immune cells in live tumours. Activatable chemokines behave as AND‐gates since they emit only after receptor binding and intracellular activation, showing enhanced selectivity over existing agents. We have applied this strategy to produce mCCL2‐MAF as the first probe for in vivo detection of metastasis‐associated macrophages in a preclinical model of lung metastasis. This strategy will accelerate the preparation of new chemokine‐based probes for imaging immune cell function in tumours.

## Graphical abstract

**Live show**: Fluorescent activatable chemokines have been designed as the first chemical probes for in vivo detection of tumour‐associated macrophages in lung metastasis. Activatable chemokines behave as AND gates since they emit only after receptor binding and intracellular activation, thereby showing enhanced selectivity over existing agents.

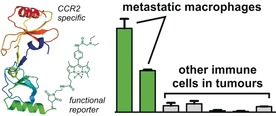

Tumour metastasis is the leading cause of cancer‐related death. The metastatic potential of tumours is largely defined by the surrounding immune environment, where macrophages are among the most abundant cells.^1^ Different subpopulations of macrophages are found in tumours, such as resident macrophages and active metastasis‐associated macrophages, with the latter being recruited by chemokines (e.g., CCL2 or C‐C motif chemokine ligand 2) to accelerate the progression of metastasis.^2^ Whereas novel therapeutic strategies to stop the recruitment of tumour‐associated macrophages—including chemokine inhibitors—are under evaluation as anticancer treatments,^3^ there are no chemical probes for detecting these subpopulations of macrophages in live tumours.

The most widespread technology to track the mobilisation of macrophages are genetically‐encoded fluorescent proteins (e.g., GFP) expressed under macrophage‐selective promoters.^4^ For instance, Entenberg et al. recently used transgenic mice to study the interactions between macrophages and cancer cells in vivo.^5^ However, this technique cannot distinguish macrophage subtypes or provide cell activity readouts. Alternatively, small‐molecule fluorophores have been developed to monitor the function of macrophages.^6^ Our group and others have shown that fluorophores responding to different biomarkers (e.g. acidic pH,^7^ reactive oxygen species,^8^ MMPs,^9^ cathepsins,^10^ and SLC transporters^11^) can be used to detect active macrophages under physiological conditions. These probes provide generic readouts of macrophage activity but are not specific for the metastasis‐associated macrophages linked to cancer progression.

CCL2 is a potent chemokine that regulates the migration of immune cells to tumours through the recognition of CCR2 receptors. Recent studies have identified that metastasis‐associated macrophages express high levels of CCR2 on the cell surface.^2^, ^12^ Binding of CCL2 to CCR2 promotes the recruitment of macrophages into metastatic sites, which accelerates the seeding and expansion of cancer cells. Given that chemokines can enter cells via their functionally‐active receptors, we hypothesized that fluorescently‐labelled CCL2 analogues would allow us to detect CCR2+ metastasis‐associated macrophages in tumours. Previously, Nibbs et al. described CCL2 chemokines to broadly identify subpopulations of mouse leukocytes.^13^ The groups of Beck‐Sickinger and Liu and Qi also demonstrated that chemically photocaged chemokines can be used to temporally control leukocyte migration.^14^ Herein, we have designed a strategy to prepare AND‐gate^15^ activatable chemokines that selectively target CCR2+ populations of metastatic macrophages in vivo.

We designed fluorescent activatable chemokines to work in a two‐step sequence (Figure 1 a). First, they recognise functional receptors that internalise upon ligand binding (step 1) AND second, they emit fluorescent signals in response to macrophage‐related activity (step 2). Compared to fluorescently‐labelled antibodies and always‐on fluorescent chemokines (Figure 2), this AND‐gate strategy makes possible the recognition of both functional receptors as well as intracellular activity, enabling the detection of highly specific cell populations. In this work, we have applied this concept to generate the first fluorescent activatable CCL2 chemokine for live imaging of active metastasis‐associated macrophages.

**Figure 1:**
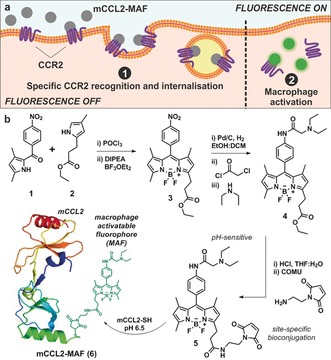
a) Schematic representation of the fluorescence activation mechanism for mCCL2‐MAF (**6**) in active CCR2+ metastasis‐associated macrophages. b) Synthesis of the BODIPY‐based macrophage‐activatable fluorophore **5** and protein conjugation to mouse chemokine CCL2 (mCCL2).

**Figure 2:**
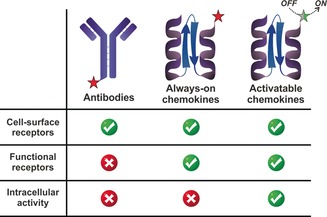
Imaging capabilities of fluorescently‐labelled antibodies, always‐on chemokines and activatable AND‐gate chemokines.

In order to conjugate the chemokine CCL2 to fluorescent markers of active macrophages, we first examined macrophage‐activatable fluorophores (MAFs) responding to intracellular metabolites associated with macrophage activity, such as phagosomal pH, superoxide and reactive oxygen species. For this, we incubated inactive and LPS‐activated RAW264.7 mouse macrophages with different MAFs and measured their emission by fluorescence microscopy (Figure S1 in the Supporting Information). We observed that pH‐sensitive BODIPY dyes triggered by phagosomal acidification showed bright intracellular fluorescence in active macrophages with the largest turn‐on emission among the different MAFs. Therefore, we decided to utilise pH‐sensitive BODIPYs for the generation of activatable CCL2 chemokines.

We synthesized the pH‐dependent BODIPY (**5**) containing a maleimide group for site‐specific conjugation to the mouse chemokine CCL2 (mCCL2, Figure 1). The key intermediate **4** was obtained in 3 steps from pyrrole **1** in good yields. As reported by our group and others,^16^ the assembling of the BODIPY fluorophore proceeded via condensation of two functionalized pyrroles (**1** and **2**, Figure 1) followed by addition of BF_3_OEt_2_ to generate the nitro‐derivatised BODIPY **3**. Herein, we optimised the catalytic hydrogenation and subsequent derivatisation with chloroacetyl chloride and diethylamine to yield BODIPY **4** with an overall yield of 87 % including all 3 steps. Finally, compound **4** was hydrolysed and coupled to 1‐(2‐aminoethyl)maleimide to generate BODIPY **5** for site‐specific protein bioconjugation (see Supporting Information for synthetic and characterisation details).

We envisaged that the incorporation of fluorophores at the C‐terminus of mCCL2 would not disrupt the recognition of chemokine receptors or impair its chemotactic activity. Therefore, we employed an analogue of mCCL2 with an extra cysteine residue at the C‐terminal end that would react site‐specifically with the BODIPY maleimide **5**. The fluorophore–chemokine conjugation was performed with an excess of compound **5** (4 equiv) in aqueous buffer at pH 6.5 for 1 h, and the resulting mCCL2‐MAF (**6**, Figure 1 b) was purified by HPLC. We used mass spectrometry to confirm single coupling of the fluorophore to the C‐terminal cysteine of mCCL2 in a 1:1 ratio (Figure S2).

Next, we evaluated the spectral properties of mCCL2‐MAF (**6**) to corroborate that the turn‐on properties of the fluorophore remained unaffected after conjugation to mCCL2. Compound **6** showed similar photophysical properties to the parent BODIPY fluorophore **4** (*λ*_exc._: 496 nm, *λ*_em._: 510 nm; Figure S3) with pH‐dependent activation and a p*K*_a_ around 4.9, suitable to detect active macrophages specifically undergoing phagosomal acidification (Figures S4 and S5). This result suggests that the incorporation of alkyl spacers at the position 3 of the BODIPY core is an effective strategy to generate bioconjugatable fluorophores with full retention of their optical properties. Next, we performed cell migration transwell assays to examine whether mCCL2‐MAF (**6**) retained the biological function of the native chemokine mCCL2. We cultured RAW264.7 mouse macrophages on top of cell‐permeable membranes, incubated them with different concentrations of mCCL2 or mCCL2‐MAF (**6**), and counted the migrated cells across the membrane. As shown in Figures 3 and S6, mCCL2‐MAF (**6**) promoted macrophage migration to comparable levels of unlabelled mCCL2. These results confirm that the C‐terminal modification of mCCL2 with BODIPY dyes endows the chemokine with fluorescent reporters of macrophage function without affecting the chemotactic properties of mCCL2.

**Figure 3:**
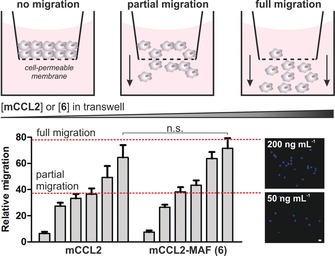
Chemotactic transwell assays in RAW264.7 macrophages. Migrated cells were quantified after incubation with culture media containing increasing levels of mCCL2 or **6** (0, 10, 25, 50, 100 and 200 ng mL^−1^) for 2 h. Cells were stained with DAPI (representative images on the right) and counted using ImageJ. Scale bar: 10 μm. Values as means ± s.d. (*n*=3). n.s. for *p*>0.05.

Given the functional and fluorescent activatable properties of mCCL2‐MAF (**6**), we then investigated its applicability as a marker for active CCR2+ metastasis‐associated macrophages. First, we compared the emission of mCCL2‐MAF (**6**) in inactive and LPS‐activated CCR2+ mouse macrophages by confocal microscopy and flow cytometry. We found that inactive macrophages showed weak fluorescence emission, which was dramatically increased in activated cells due to LPS‐induced phagosomal acidification (Figures 4 a,b). Importantly, the fluorescence enhancement was reduced to basal levels when macrophages were pre‐treated with bafilomycin A, which inhibits LPS‐mediated phagosomal acidification, or anti‐CCR2 antibodies, which block ligand binding to the receptor and subsequent endocytosis (Figure 4 c). Altogether these results confirm that the fluorescence emission of mCCL2‐MAF (**6**) follows an activation mechanism with two sequential events: 1) CCR2‐mediated internalisation, and 2) fluorescence amplification in active macrophages.

**Figure 4:**
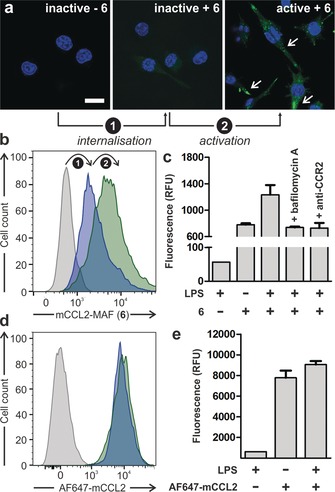
Fluorescence microscopy (a) and flow cytometric analysis (b–e) of inactive and active CCR2+ mouse macrophages upon treatment with LPS (100 ng mL^−1^ for 18 h), compound **6** (100 nm, b,c), and AF647‐mCCL2 (100 nm, d,e). Cells were counterstained with Hoechst 33 342 for nuclear staining. In panel (a) white arrows point at fluorescent acidic phagosomes inside macrophages. Scale bar: 10 μm. Values are presented as means ± s.d. (*n*=3).

In contrast to mCCL2‐MAF (**6**), AlexaFluor647‐mCCL2 (always‐on chemokine) emitted strong fluorescence in CCR2+ mouse macrophages regardless of their activation state (Figure 4 d), which highlights the advantages of activatable chemokines as fluorescent reporters of intracellular activity.

Encouraged by these results, we next examined whether mCCL2‐MAF (**6**) could selectively stain CCR2+ macrophages in the presence of other immune cells. For these experiments, we used wild‐type C57/BL6 as well as *Ccr2* knock‐out (KO) mice so that we could determine the selectivity profile of compound **6** in cell populations expressing variable levels of CCR2 receptors. We prepared single‐cell suspensions from spleens of wild‐type and *Ccr2* KO mice, incubated them with mCCL2‐MAF (**6**) (100 nm) and analysed them by flow cytometry. We employed mouse antibodies (F4/80, CD45, CD11b, Ly6G, CD3 and CD19) to identify different immune cells,^17^ which included macrophages, neutrophils, B cells and T cells (Figure S7 for gating strategy). From this analysis, we found that a subpopulation of wild‐type macrophages showed bright fluorescence whereas this cell population was not detected in macrophages from *Ccr2* KO mice (Figure 5 a), indicating that the signals from mCCL2‐MAF (**6**) are CCR2‐dependent. We also found that mCCL2‐MAF (**6**) did not stain neutrophils, T cells or B cells in either wild‐type of KO mice (Figure 5 b). These results highlight the value of mCCL2‐MAF (**6**) for the selective detection of CCR2+ macrophages with minimal off‐target fluorescence in other cells.

**Figure 5:**
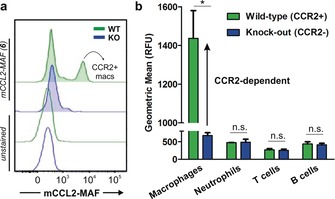
Ex vivo staining profile of compound **6** (100 nm) in different immune cells from spleens of wild‐type and *Ccr2* KO mice. a) Representative fluorescence histograms of compound **6**‐stained and unstained macrophages from wild‐type mice (green) and *Ccr2* KO (blue) mice (*n*=3 for each group). b) Geometric fluorescence intensity of immune cells: macrophages (Ly6G^−^F4/80^+^CD11b^+^), neutrophils (Ly6G^+^F4/80^−^), T cells (CD3^+^CD19^−^), and B cells (CD3^−^CD19^+^). Values as means ± s.d. (*n*=3). * for *p*<0.05, n.s. for *p*>0.05.

Finally, we examined whether mCCL2‐MAF (**6**) could detect metastasis‐associated macrophages in vivo. To this end, we used a mouse model of lung metastasis where E0771‐LG mouse mammary tumour cells are injected into the tail vein of syngeneic C57BL/6 mice to mimic the systemic distribution of cancer cells. In this model, metastasis‐associated macrophages (MAMs) and their progenitor cells (MAMPCs) accumulate in the metastatic lungs via CCL2‐CCR2 signaling.^17^, ^18^ Since the accumulation of MAMs and MAMPCs promotes metastasis, these cells are regarded as attractive targets in anticancer therapy.^17^, ^18^, ^19^ Animals injected with E0771‐LG cancer cells formed metastatic tumours in their lungs after 2 weeks, as confirmed by whole‐body bioluminescence imaging (Figure S8). At this point, we injected mCCL2‐MAF (**6**) via the tail vein (10 ng in 50 μL saline) and harvested the metastatic lungs after 2 hours. Histological examination of the lungs corroborated the presence of metastatic nodules (Figure 6 a). Notably, in the metastatic nodules, we found F4/80+ macrophages showing green fluorescence (Figure 6 b), which indicates that the in vivo injection of mCCL2‐MAF (**6**) labels a defined subpopulation of macrophages in metastatic tumours. We also analysed the tissues by flow cytometry and measured the fluorescence signals in major immune cells found in metastatic lungs (Figure S9 for gating strategy and immune cell markers). As shown in Figure 6 c, we found high levels of green fluorescence in active MAMPCs and MAMs that require CCR2 signaling for their accumulation in metastatic sites. Remarkably, all other immune cells in the metastatic lungs [for example, resident macrophages (RMAC), neutrophils, T cells, B cells, natural killer (NK cells)] remained unstained by compound **6** (Figure 6 c).

**Figure 6:**
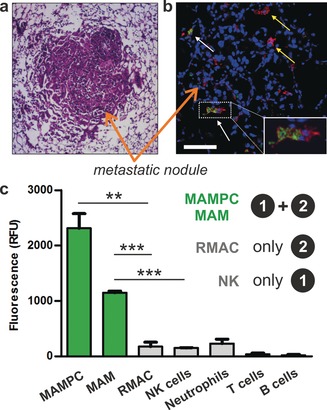
In vivo characterisation of metastatic lungs after tail vein injection of mCCL2‐MAF (**6**). a) Histological H&E staining of metastatic nodules from lungs of tumour‐bearing mice. b) Confocal microscopy images of metastatic lungs (blue: Hoeschst 33342 for nuclei; green: compound **6**, red: anti‐F4/80 for all macrophages). White arrows point at **6**‐stained MAMs and yellow arrows point at **6**‐unstained RMACs. Scale bar: 100 μm. c) Fluorescence profiling of immune cells after in vivo staining with compound **6** (Figure S9 for gating strategy). Values presented as means ± s.d. (*n*=3).** for *p*<0.01, *** for *p*<0.001.

These results confirmed that the AND‐gate activation mechanism of mCCL2‐MAF (**6**) was recapitulated in vivo. Specifically, we did not observe staining in RMAC, which do not express CCR2, or in NK cells, which express high levels of CCR2 but cannot trigger the emission of macrophage‐activated fluorophores (Figure 6 c). In contrast, the always‐on chemokine AF647‐mCCL2 showed bright fluorescence in NK cells from metastatic tumours under the same experimental conditions (Figure S10). This observation confirms that the combination of functional chemokines with activatable fluorophores can generate highly specific probes for immune cells that cannot be targeted with conventional reagents, and validates mCCL2‐MAF (**6**) as the first chemical probe for selective detection and imaging of active metastasis‐associated macrophages in tumours in vivo.

In summary, we have designed AND‐gate activatable chemokines as fluorescent agents for imaging active subpopulations of immune cells in live tumours. We have synthesized mCCL2‐MAF as the first chemical probe for metastasis‐associated macrophages by coupling a Cys‐modified chemokine CCL2 with a thiol‐reactive macrophage‐activatable fluorophore. mCCL2‐MAF fully retains chemotactic activity and emits bright intracellular fluorescence in a CCR2‐ AND macrophage activity‐dependent manner. We also demonstrated the utility of our strategy by showing selective staining of metastasis‐associated macrophages in vivo, outperforming always‐on fluorescent chemokines. This chemical strategy will accelerate the design of highly specific chemokine‐based imaging agents to study immune cell subpopulations with enhanced spatial and temporal resolution.

## Conflict of interest

The authors declare no conflict of interest.

## Supporting information

As a service to our authors and readers, this journal provides supporting information supplied by the authors. Such materials are peer reviewed and may be re‐organized for online delivery, but are not copy‐edited or typeset. Technical support issues arising from supporting information (other than missing files) should be addressed to the authors.

Supplementary
