# Supplementary material for: A Fluorescent Activatable AND‐Gate Chemokine CCL2 Enables In Vivo Detection of Metastasis‐Associated Macrophages
Source: Angew Chem Int Ed Engl. 2019 Oct 11;58(47):16894–8. doi: 10.1002/anie.201910955 (PMC6900180; doi:10.1002/anie.201910955)
Supplement: Supplementary file 1 — Supplementary [file ANIE-58-16894-s001.pdf]

Angewandte Chemie
Eine Zeitschrift der Gesellschaft Deutscher Chemiker

Supporting Information

A Fluorescent Activatable AND-Gate Chemokine CCL2 Enables In Vivo Detection of Metastasis-Associated Macrophages

Antonio Fernandez, Emily J. Thompson, Jeffrey W. Pollard, Takanori Kitamura, and Marc Vendrell**

anie_201910955_sm_miscellaneous_information.pdf

Electronic Supporting Information

Table of Contents

1. Chemical Synthesis.

2. NMR Spectra.

3. Supplementary Figures.

4. Experimental Protocols for Spectroscopy and Biological Assays.

5. Supplementary References.

Chemical synthesis

General materials.

Commercially available reagents were used without further purification. All mCCL2 analogues were purchased from Almac. Thin-layer chromatography was conducted on Merck silica gel 60 F254 sheets and visualized by UV (254 and 365 nm). Silica gel (particle size 35−70 μm) was used for column chromatography. ^{1}H and ^{13}C NMR spectra were recorded in a Bruker Avance 500 spectrometer (at 500 and 126 MHz, respectively). Data for ^{1}H NMR spectra are reported as chemical shift δ (ppm), multiplicity, coupling constant (Hz), and integration. Data for ^{13}C NMR spectra are reported as chemical shifts relative to the solvent peak. HPLC−MS analysis was performed on a Waters Alliance 2695 separation module connected to a Waters PDA2996 photo-diode array detector and a ZQ Micromass mass spectrometer (ESI-MS) with a Phenomenex column (C_{18}, 5 μm, 4.6 × 150 mm). Chemokine conjugates were purified using a Waters HPLC system using a Phenomenex column (C_{18}, 5 μm, 4.6 × 150 mm) and UV detection.

3,5-dimethyl-1H-pyrrol-2-yl)-(4-nitrophenyl)methanone (1).

$POCl_3$ (430 μL, 4.6 mmol) was added to *N,N*-dimethyl-4-nitro-benzamide (300 mg, 1.54 mmol) under N_2 atmosphere. The mixture was stirred at 25˚C for 6 h. Then, a solution of 2,4-dimethylpyrrole (159 μL, 1.54 mmol) in dichloroethane (5 mL) was added. After 18 h, the crude reaction was poured into 15 mL of a 10% aqueous solution of K_2CO_3, stirred at r.t. for 15 min and at 85˚C for another 45 min. The crude reaction was extracted with CH_2Cl_2 and the combined organic extracts were dried over anhydrous $MgSO_4$. Finally, the solvent was removed under reduced pressure and the crude was recrystallised in Et_2O to obtain compound **1** as a yellow solid (51% yield).

^{1}H NMR (500 MHz, MeOD) δ 8.38 (d, J = 8.8 Hz, 2H), 7.81 (d, J = 8.9 Hz, 2H), 5.96 (t, J = 0.8 Hz, 1H), 5.52 (s, 1H), 2.30 (s, 3H), 2.02 (s, 3H).

^{13}C NMR (126 MHz, MeOD) δ 184.7, 150.5, 147.6, 139.6, 134.4, 130.2, 128.3, 124.7, 114.5, 66.9, 14.2, 12.8.

MS (m/z): $[M+H]^+$ calcd. for $C_{13}H_{13}N_2O_3^+$: 245.1; found: 245.3.

Ethyl 3-(4-methyl-1*H*-pyrrol-2-yl)propanoate (2).

4-methyl-2-pyrrolecarboxaldehyde (310 mg, 2.84 mmol) and carbethoxymethylene (1.44 g, 4.3 mmol) were dissolved in 15 mL of benzene and stirred under reflux overnight. Then, the solvent was removed under reduced pressure and the mixture was purified by normal phase chromatography (Hexane: EtOAc, 48:2) to obtain 510 mg of ethyl (*E*)-3-(4-methyl-1*H*-pyrrol-2-yl)-2-propenoate as a white solid (99% yield). Then, Pd/C (51 mg, 10% mol) was added to a solution of ethyl (*E*)-3-(4-methyl-1*H*-pyrrol-2-yl)-2-propenoate (510 mg, 2.85 mmol) in 20 mL of EtOH. The resulting mixture was degassed and stirred under H_2 (1 atm) at r.t. for 12 h. When the reaction was completed, inorganic solids were removed by filtration through Celite® and washed with EtOH (3 × 10 mL). The solvent was removed under reduced pressure affording compound **2** as yellowish oil (98% yield).

^{1}H NMR (500 MHz, MeOD) δ 6.36 (s, 1H), 5.66 (s, 1H), 4.15 (q, *J* = 7.1 Hz, 2H), 2.84 (t, *J* = 7.5 Hz, 2H), 2.60 (t, *J* = 7.5 Hz, 2H), 2.03 (s, 3H), 1.26 (t, *J* = 7.1 Hz, 3H).

^{13}C NMR (126 MHz, MeOD) δ 175.0, 131.6, 118.8, 115.2, 107.2, 61.5, 35.7, 24.1, 14.5, 12.1.

MS (m/z): $[M+H]^+$ calcd. for $C_{10}H_{16}NO_2^+$: 182.1; found: 182.2.

Ethyl 3-(5,5-difluoro-1,7,9-trimethyl-10-(4-nitrophenyl)-5H-4 λ^4, 5 λ^4-dipyrrolo[1,2-c:2',1'-f][1,3,2]diazaborinin-3-yl)propanoate (3).

To a solution of compound **1** (216 mg, 0.88 mmol) and compound **2** (100 mg, 0.55 mmol) in CH_2Cl_2 (15 mL), $POCl_3$ (103 µL, 1.1 mmol) was added at 0˚C. The mixture was stirred overnight at r.t. Then, DIPEA (385 µL, 2.2 mmol) was added dropwise and after 10 min, $BF_3 \cdot OEt_2$ (689 µL, 2.2 mmol) was added in one portion. The reaction was stirred for 2 h and all the volatiles were evaporated under reduced pressure. The crude mixture was purified by normal phase chromatography (Hexane: EtOAc, 9:1) to obtain 110 mg of compound **13** as a red solid (44% yield).

^{1}H NMR (500 MHz, $CDCl_3$) δ 8.41 (d, *J* = 8.7 Hz, 2H), 7.57 (d, *J* = 8.7 Hz, 2H), 6.09 (s, 1H), 6.06 (s, 1H), 4.18 (q, *J* = 7.1 Hz, 2H), 3.31 (t, *J* = 7.5 Hz, 2H), 2.77 (t, *J* = 7.5 Hz, 2H), 2.59 (s, 3H), 1.39 (s, 3H), 1.39 (s, 3H), 1.29 (t, *J* = 7.2 Hz, 3H).

^{13}C NMR (126 MHz, $CDCl_3$) δ 172.4, 158.1, 157.8, 148.4, 143.3, 142.2, 141.9, 138.8, 130.4, 129.6, 124.4, 122.3, 122.3, 120.3, 120.3, 60.6, 33.2, 23.8, 14.8, 14.8, 14.2.

MS (m/z): $[M+Na]^+$ calcd. for $C_{23}H_{24}BF_2N_3NaO_4^+$*:* 478.2; found: 478.4.

Ethyl 3-(10-(4-(2-(diethylamino)acetamido)phenyl)-5,5-difluoro-1,7,9-trimethyl-5*H*-4l4,5l4-dipyrrolo[1,2-c:2',1'-f][1,3,2]diazaborinin-3-yl)propanoate (4).

To a solution of compound **3** (100 mg, 0.21 mmol) in 16 mL of degassed EtOH:CH_2Cl_2 (1:1), was added a suspension of Pd/C (10 mg, 10% mol) in EtOH under inert atmosphere. The resulting mixture was stirred at r.t. under H_2 (1 atm) for 12 h. When the reaction was completed, inorganic solids were removed by filtration through Celite® and washed with several portions of CH_2Cl_2. The solvent was removed under reduced pressure affording 89 mg of amino-BODIPY as an orange solid (quantitative yield). Then, to a solution of amino-BODIPY in ACN (10 mL) was added chloroacetyl chloride (25 µL, 0.32 mmol) at 0˚C. The reaction was stirred at r.t. for 4 h and diethylamine (32 µL, 0.32 mmol) was added dropwise. The reaction was stirred overnight, and all the volatiles were evaporated under reduced pressure. The crude mixture was purified by normal phase chromatography (CH_2Cl_2: MeOH, 95:5) to obtain 99 mg of compound **4** as an orange solid (87% yield).

^{1}H NMR (500 MHz, $CDCl_3$) δ 7.83−7.67 (m, 2H), 7.26 (d, *J* = 8.4 Hz, 2H), 6.03 (d, *J* = 14.4 Hz, 2H), 4.29−4.09 (m, 2H), 3.30 (t, *J* = 7.5 Hz, 2H), 3.21 (br s, 4H), 2.75 (m, 6H), 2.57 (s, 3H), 1.47 (s, 3H), 1.46 (s, 3H), 1.28 (t, *J* = 7.1 Hz, 3H), 1.15 (br s, 6H).

^{13}C NMR (126 MHz, CDCl3) δ = 172.6, 156.9, 156.5, 143.9, 142.8, 141.9, 138.6, 132.0, 131.4, 128.7, 121.6, 119.6, 60.5, 48.7, 33.3, 23.8, 23.7, 14.8, 14.7, 14.2.

MS (m/z): $[M+H]^+$ calcd. for $C_{29}H_{38}BF_2N_4O_3^+$: 539.3; found: 539.3.

3-(10-(4-(2-(diethylamino)acetamido)phenyl)-5,5-difluoro-1,7,9-trimethyl-5H-4l4,5l4-dipyrrolo[1,2-c:2',1'-f][1,3,2]diazaborinin-3-yl)-N-(2-(2,5-dioxo-2,5-dihydro-1H-pyrrol-1-yl)ethyl)propenamide (5).

To a solution of compound **4** (58 mg, 0.11 mmol) in THF (3 mL) were added H_2O (2 mL) and conc. HCl (1 mL). The reaction mixture was stirred at r.t. for 24 h. Then, the crude mixture was extracted with CH_2Cl_2 (4 × 15 mL), the organic extracts were dried over $MgSO_4$, filtered and evaporated under reduced pressure. Finally, the mixture was purified by column chromatography (CH_2Cl_2: MeOH, 45:5) to obtain 30 mg of carboxylic acid BODIPY as an orange solid (54% yield). Then, carboxylic acid BODIPY (16 mg, 0.03 mmol) and COMU (20 mg, 0.06) were dissolved in DMF: CH_2Cl_2 (500 µL, 1:1) and DIPEA (9 µL, 0.06 mmol). After 5 min stirring at r.t. *N*-(2-aminoethyl)maleimide trifluoroacetate salt (16 mg, 0.09 mmol) was added in DMF: CH_2Cl_2 (200 µL, 1:1) and DIPEA (18 µL, 0.09 mmol). The crude mixture was purified by reverse phase semi-preparative HPLC to obtain 11 mg of compound **5** as an orange solid (58% yield).

^{1}H NMR (500 MHz, MeOD) δ 7.85 (d, *J* = 8.5 Hz, 2H), 7.35 (d, *J* = 8.5 Hz, 2H), 6.82 (s, 2H), 6.12 (d, *J* = 4.7 Hz, 2H), 4.39 (q, *J* = 7.2 Hz, 4H), 4.09 (s, 2H), 3.72 – 3.57 (m, 2H), 3.45 – 3.33 (m, 2H), 2.96 – 2.66 (m, 2H), 2.54 (s, 3H), 2.52 (d, *J* = 3.0 Hz, 2H), 1.48 (s, 6H), 1.43 – 1.33 (m, 6H).

^{13}C NMR (126 MHz, MeOD) δ 173.5, 171.1, 159.0, 138.7, 134.0, 134.0, 130.9, 128.7, 125.9, 121.3, 120.1, 107.8, 62.5, 54.0, 37.5, 36.9, 34.3, 23.8, 13.5, 13.4, 12.9, 8.2.

HRMS (m/z): $[M+H]^+$ calcd. for $C_{33}H_{39}BF_2N_6O_4^+$: 633.3134; found: 633.3166.

mCCL2-MAF (6).

10 µL of compound **5** (0.3 mg mL^{-1}) in DMSO were added to mCCL2-thiol (100 µg, 0.011 µmol) in phosphate buffer (90 µL, pH 6.5). The mixture was stirred a r.t. for 1 h in the dark and the crude was purified by reverse phase HPLC [eluents: H_2O-ACN (0.1% HCOOH)] to obtain 24 µg (determined by UV absorption) of compound **6** as an orange solid (24% yield).

MS-MALDI (m/z): $[M+2Na]^+$ calcd.: 9561.0, found: 9561.6.

NMR spectra

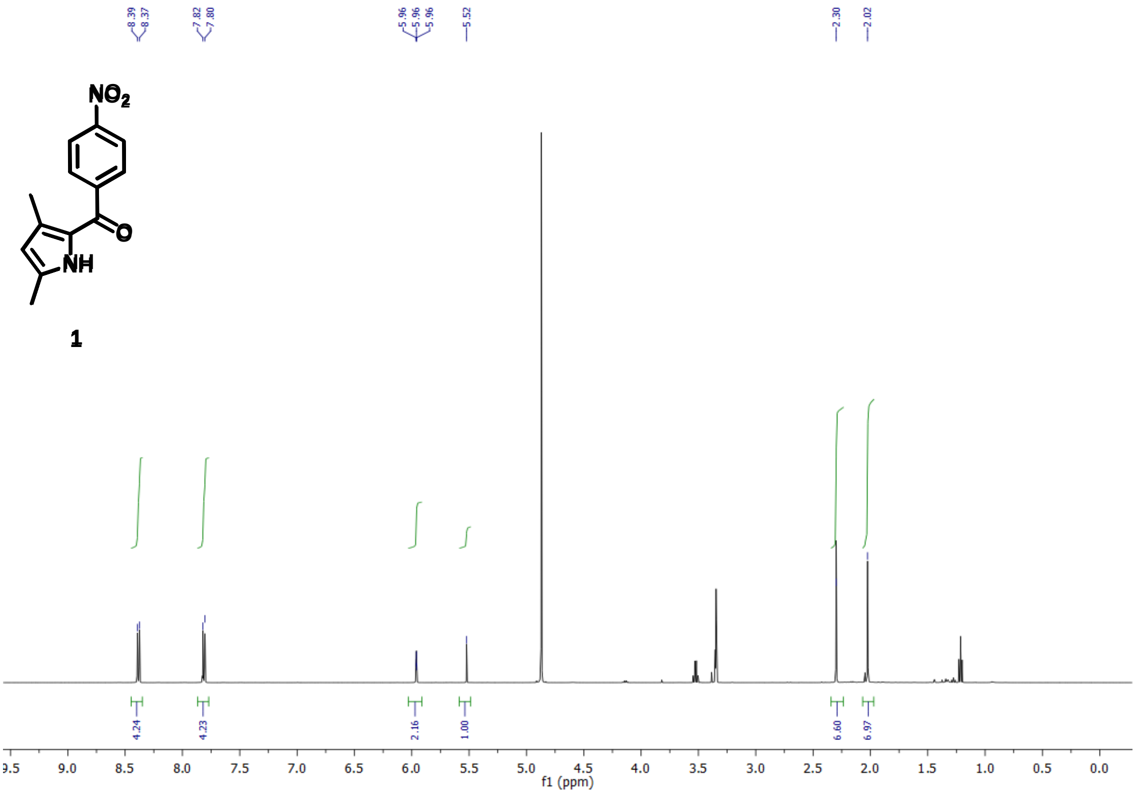
8.39
8.37
7.82
7.80
5.96
5.96
5.96
5.52
2.30
2.02
NO2
O
NH
1
4.24
4.23
2.16
1.00
6.60
6.97
f1 (ppm)

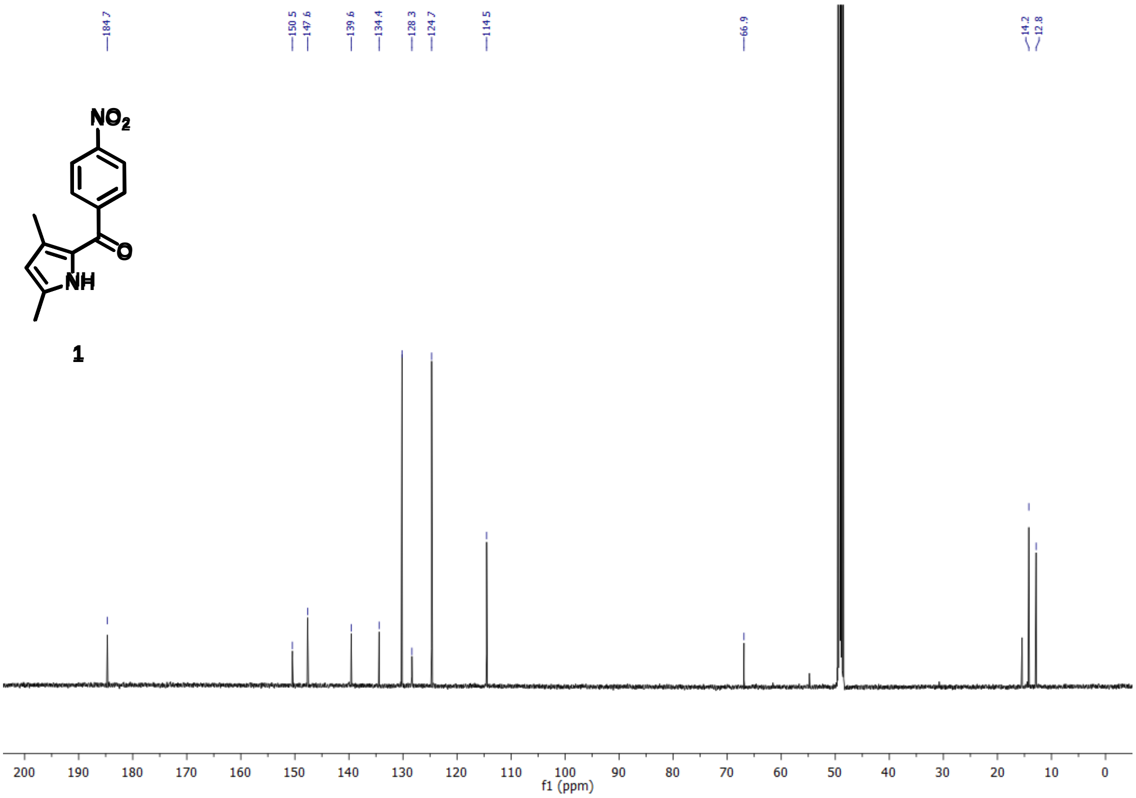
184.7
150.5
147.6
139.6
134.4
128.3
124.7
114.5
66.9
14.2
12.8
NO2
O
NH
1
f1 (ppm)

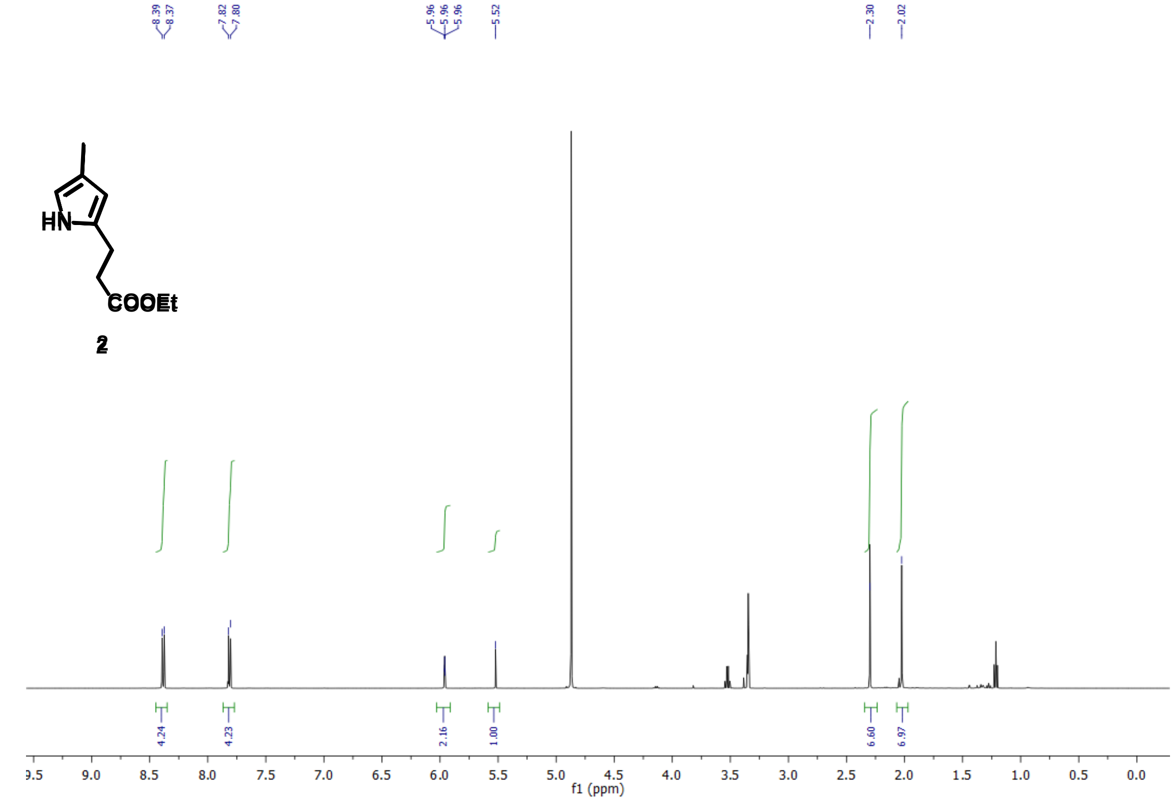
8.39
8.37
7.82
7.80
5.96
5.96
5.96
5.52
2.30
2.02
HN
COOEt
2
4.24
4.23
2.16
1.00
6.60
6.97
f1 (ppm)

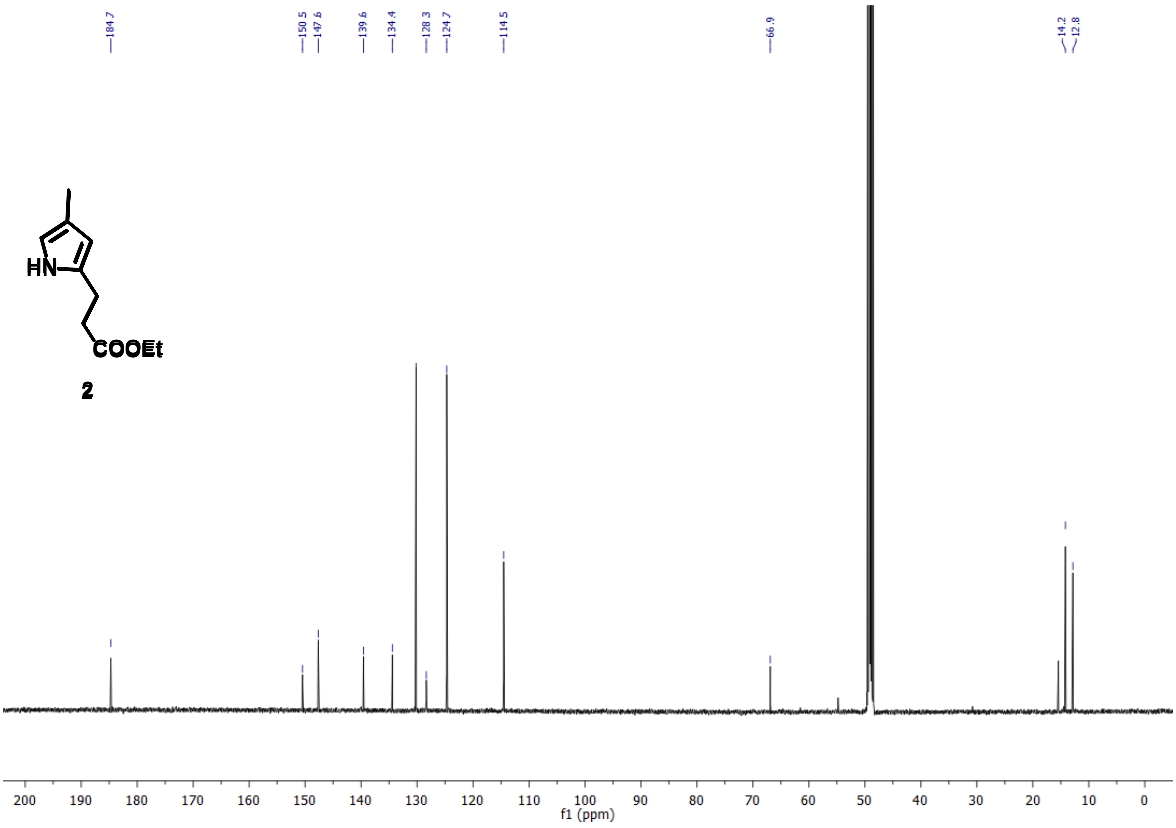
184.7
150.5
147.6
139.6
134.4
128.3
124.7
114.5
66.9
14.2
12.8
HN
COOEt
2
f1 (ppm)

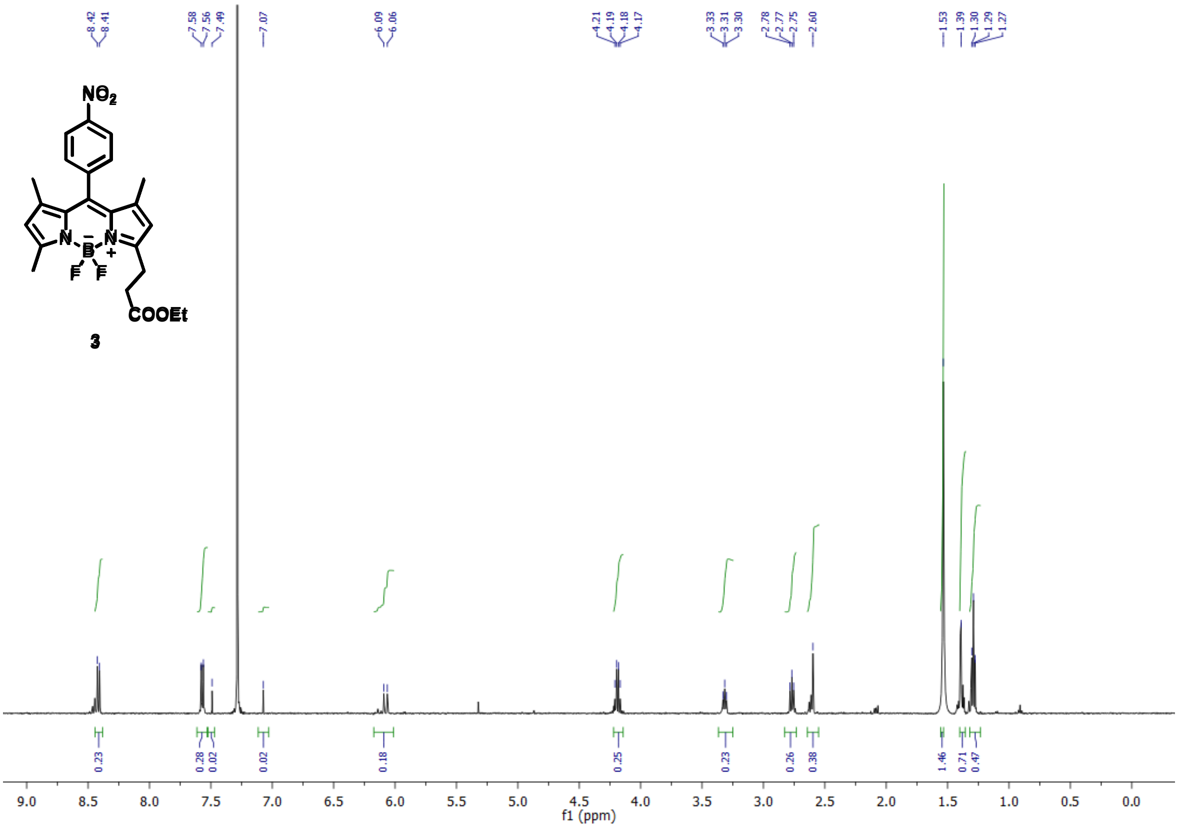
8.42
8.41
7.58
7.56
7.49
7.07
6.09
6.06
4.21
4.19
4.18
4.17
3.33
3.31
3.30
2.78
2.77
2.75
2.60
1.53
1.39
1.30
1.29
1.27
NO2
N
B
N
F
F
COOEt
3
0.23
0.28
0.02
0.02
0.18
0.25
0.23
0.26
0.38
1.46
0.71
0.47
9.0 8.5 8.0 7.5 7.0 6.5 6.0 5.5 5.0 4.5 4.0 3.5 3.0 2.5 2.0 1.5 1.0 0.5 0.0
f1 (ppm)

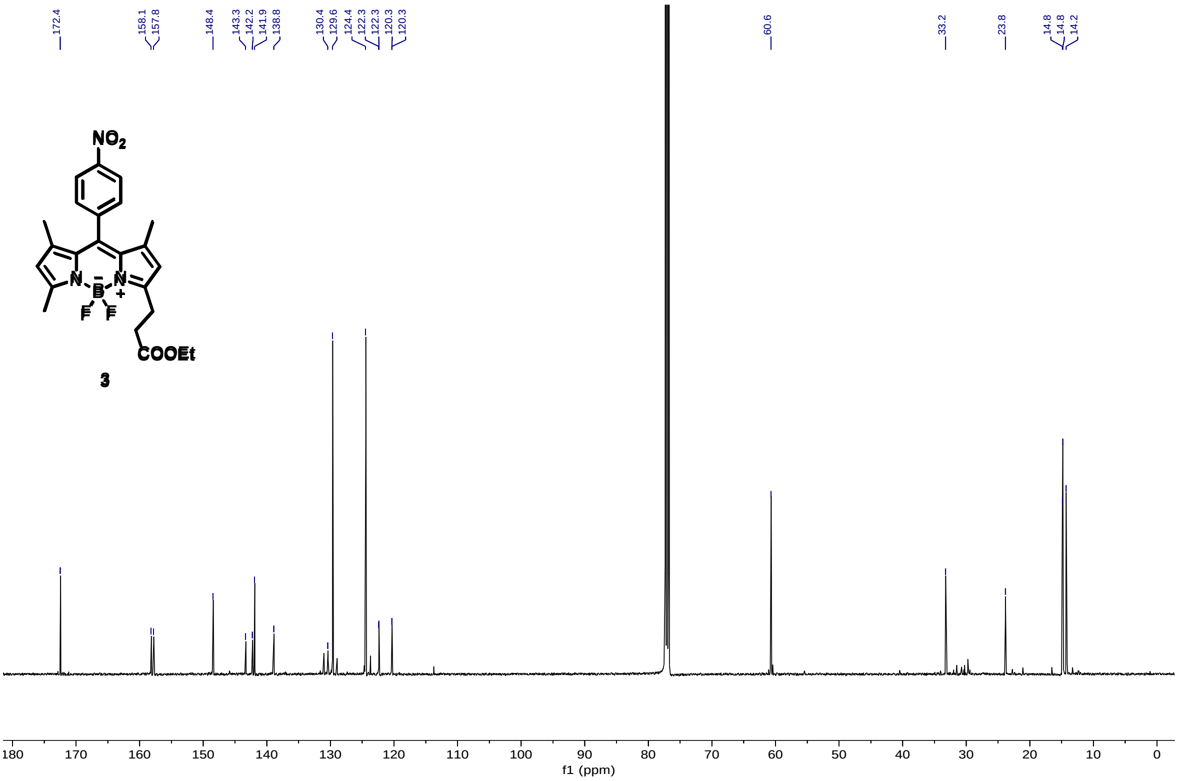
172.4
158.1
157.8
148.4
143.3
142.2
141.9
138.8
130.4
129.6
124.4
122.3
122.3
120.3
120.3
60.6
33.2
23.8
14.8
14.8
14.2
NO2
N
B
N
F
F
COOEt
3
180 170 160 150 140 130 120 110 100 90 80 70 60 50 40 30 20 10 0
f1 (ppm)

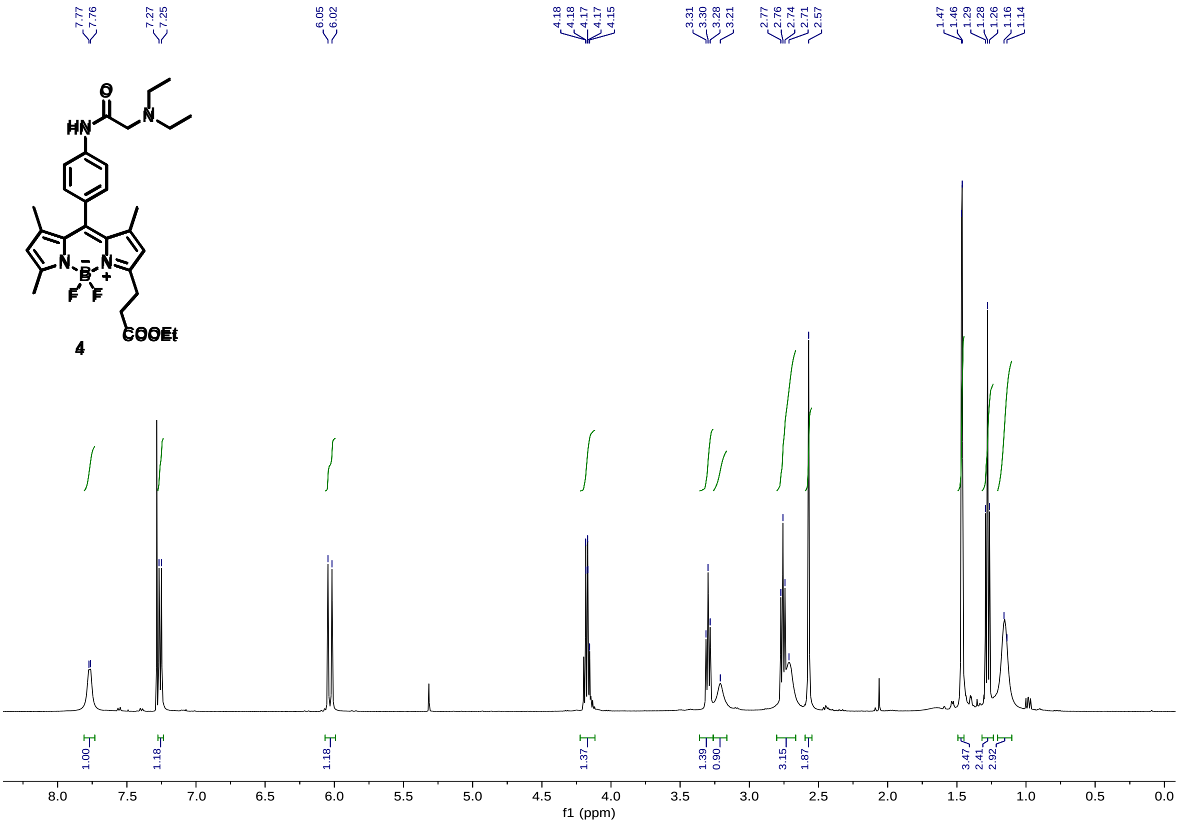
7.77
7.76
7.27
7.25
6.05
6.02
4.18
4.18
4.17
4.17
4.15
3.31
3.30
3.28
3.21
2.77
2.76
2.74
2.71
2.57
1.47
1.46
1.29
1.28
1.26
1.16
1.14
HN
O
N
N
B
N
F F
+
COOEt
4
1.00
1.18
1.18
1.37
1.39
0.90
3.15
1.87
3.47
2.41
2.92
8.0 7.5 7.0 6.5 6.0 5.5 5.0 4.5 4.0 3.5 3.0 2.5 2.0 1.5 1.0 0.5 0.0
f1 (ppm)

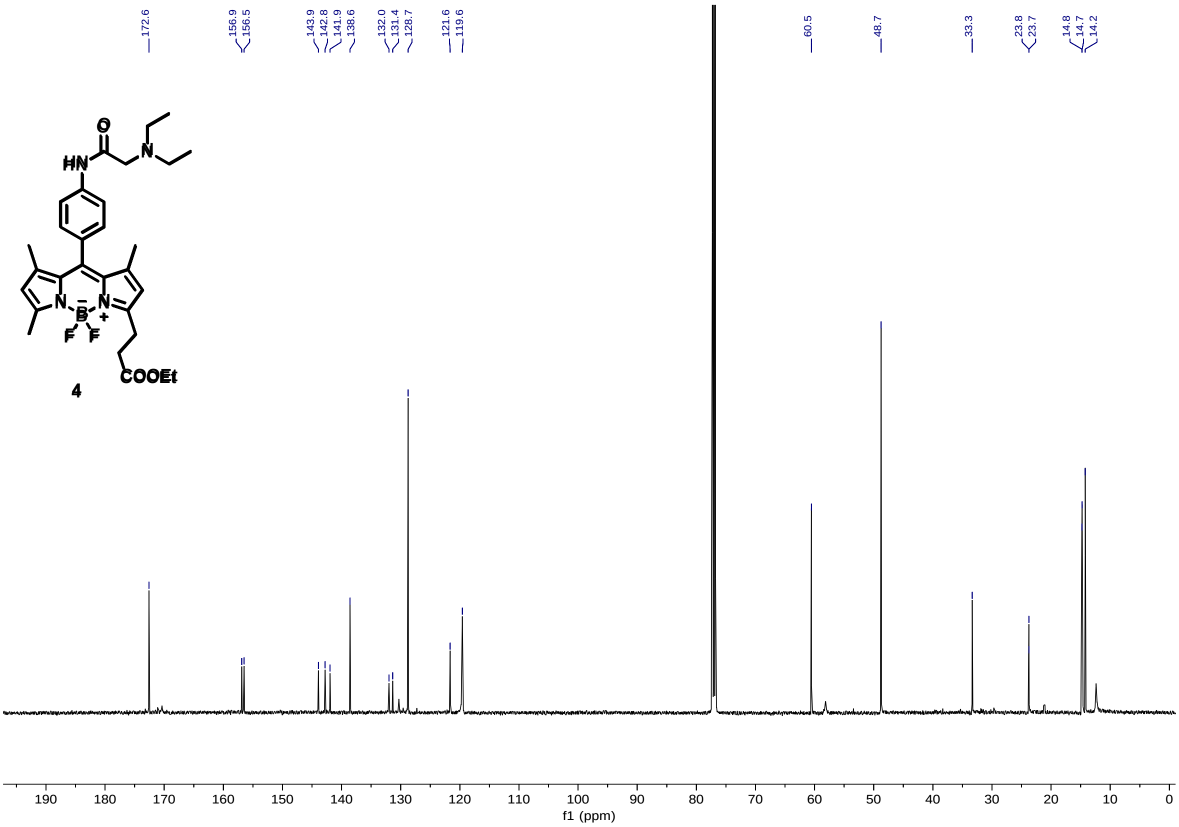
172.6
156.9
156.5
143.9
142.8
141.9
138.6
132.0
131.4
128.7
121.6
119.6
60.5
48.7
33.3
23.8
23.7
14.8
14.7
14.2
HN
O
N
N
B
N
F F
+
COOEt
4
190 180 170 160 150 140 130 120 110 100 90 80 70 60 50 40 30 20 10 0
f1 (ppm)

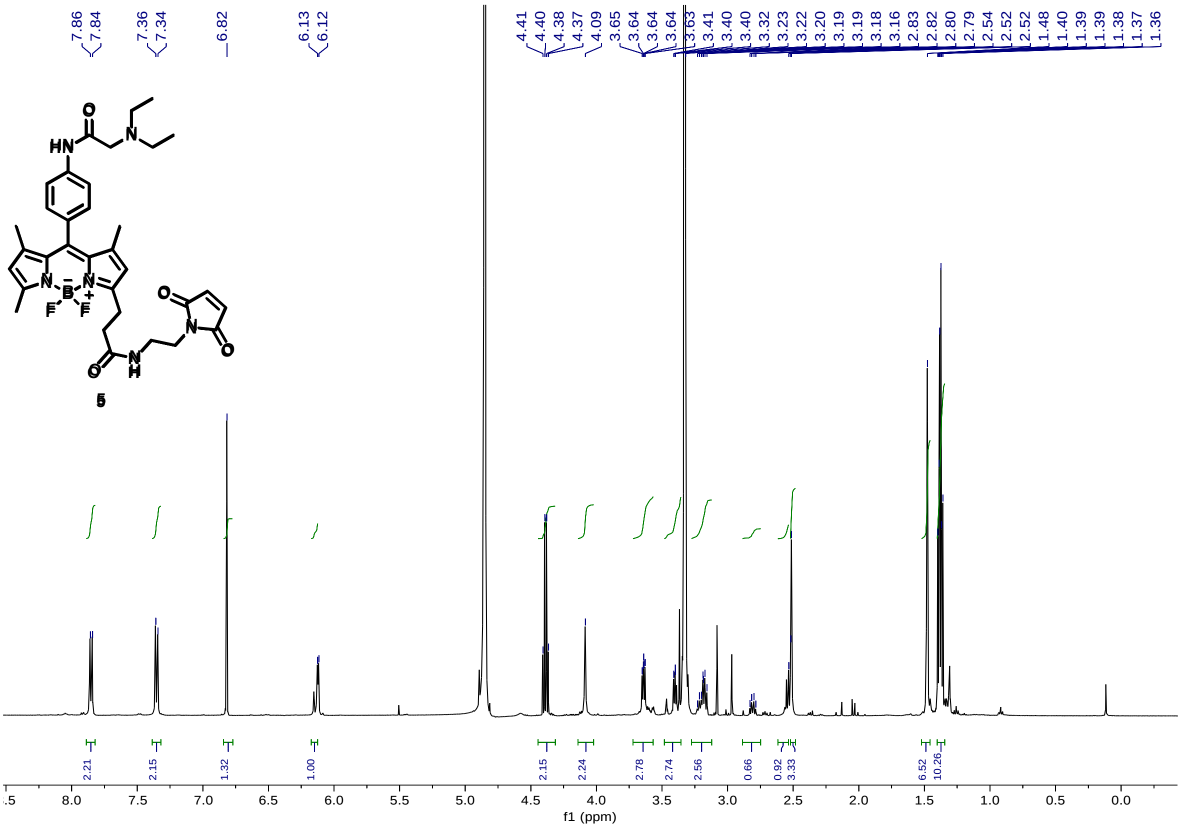
7.86 7.84 7.36 7.34 6.82 6.13 6.12 4.41 4.40 4.38 4.37 4.09 3.65 3.64 3.64 3.64 3.63 3.41 3.40 3.40 3.32 3.23 3.22 3.20 3.19 3.19 3.18 3.16 2.83 2.82 2.80 2.79 2.54 2.52 2.52 1.48 1.40 1.39 1.39 1.38 1.37 1.36
2.21 2.15 1.32 1.00 2.15 2.24 2.78 2.74 2.56 0.66 0.92 3.33 6.52 10.26
5
f1 (ppm)

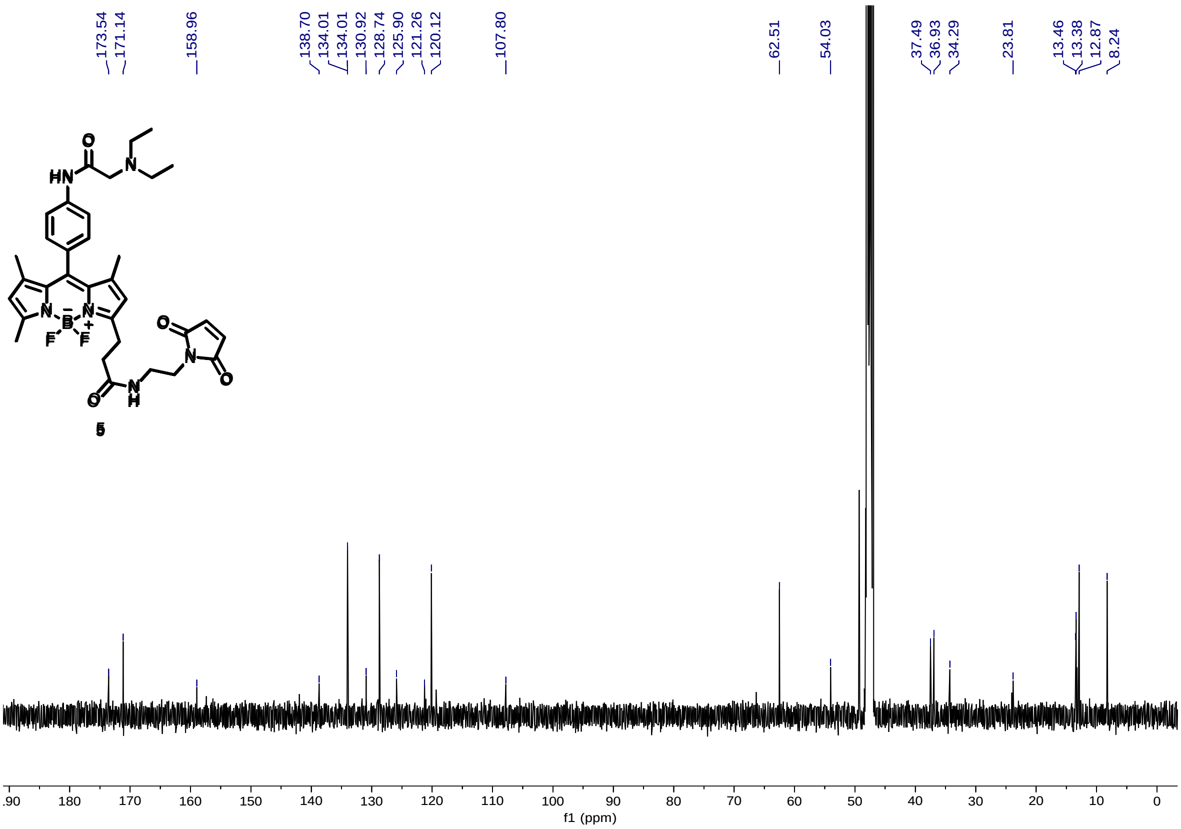
173.54 171.14 158.96 138.70 134.01 134.01 130.92 128.74 125.90 121.26 120.12 107.80 62.51 54.03 37.49 36.93 34.29 23.81 13.46 13.38 12.87 8.24
5
f1 (ppm)

Supplementary Figures

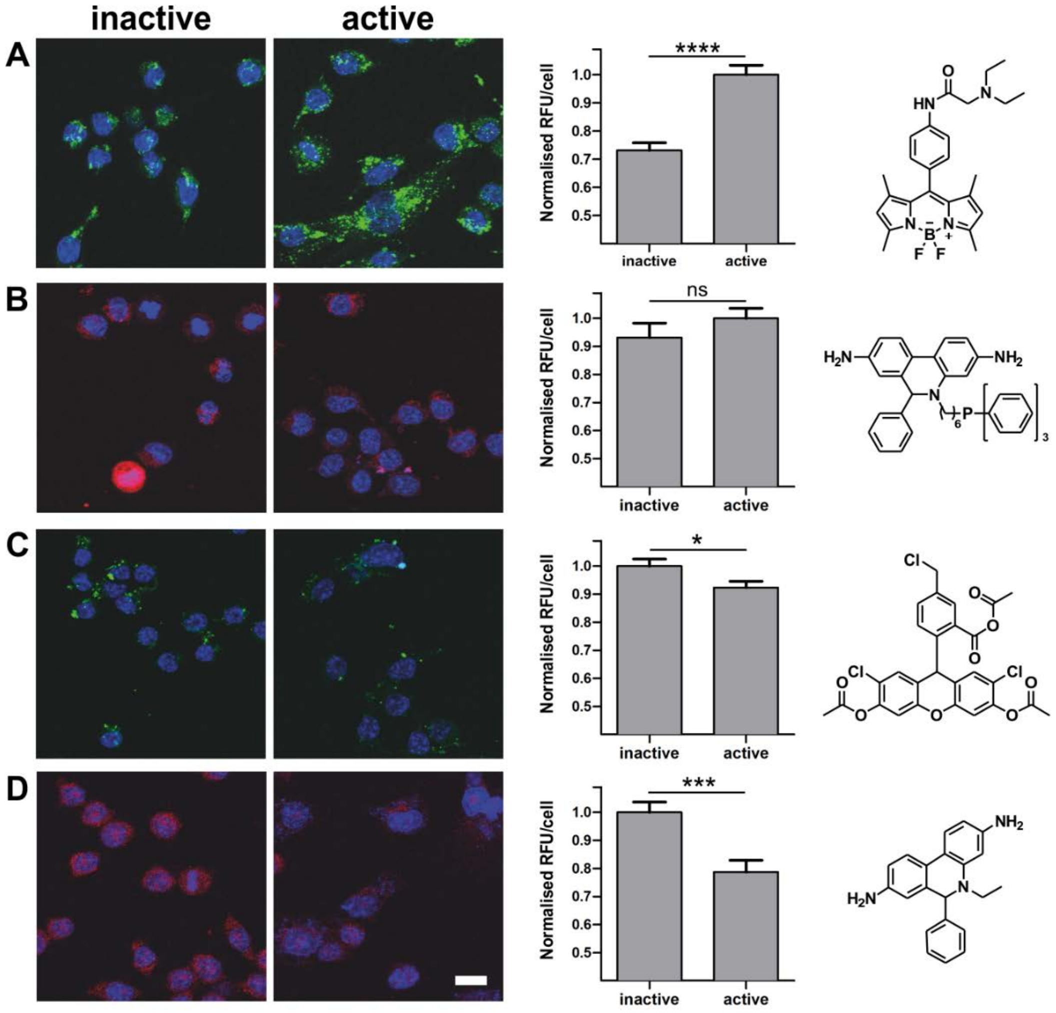

Figure S1. Image-based screening of fluorophores responding to different intracellular metabolites associated to macrophage activity. Inactive (*left panels*) and LPS-activated (100 ng mL^{-1} for 18 h, *right panels*) RAW264.7 mouse macrophages were incubated with fluorophores responding to: A) phagosomal pH (PhagoGreen[1], 100 nM), B) mitochondrial superoxide (MitoSOX[2], 5 μM), C) general oxidative stress indicator (CM-H2DCFDA[3], 5 μM), D) intracellular superoxide (dihydroethidium[4], 10 μM). Fluorescence images were analysed and quantified by ImageJ. Values are presented as means±s.e.m. (n=3). Scale bar: 10 μm. ns for $p > 0.05$, * for $p < 0.05$, *** for $p < 0.001$, **** for $p < 0.0001$.

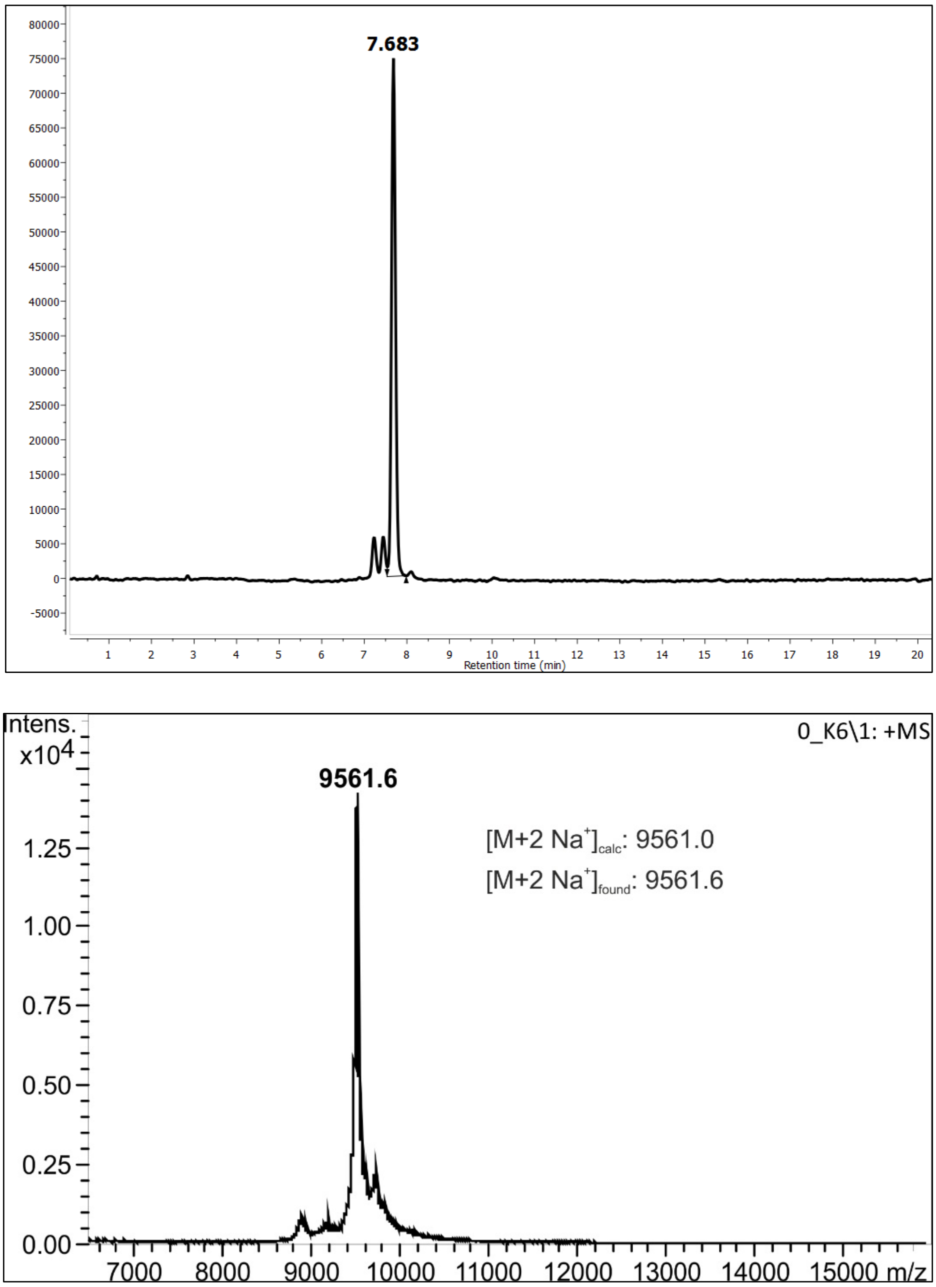

Figure S2. HPLC trace (UV detection: 280 nm, top panel) and MALDI spectrum (bottom panel) of purified mCCL2-MAF (**6**). Mw (mCCL2-thiol): 8884.5, Mw (compound **5**): 632.5.

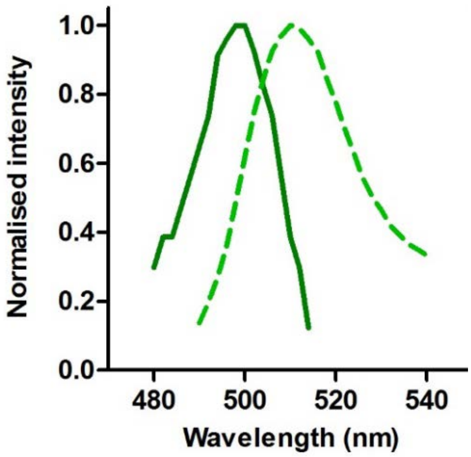

Figure S3. Spectral characterisation of mCCL2-MAF (**6**). Absorbance (solid line) and emission (dashed line) spectra measured in PBS. $\lambda_{exc.}$: 460 nm.

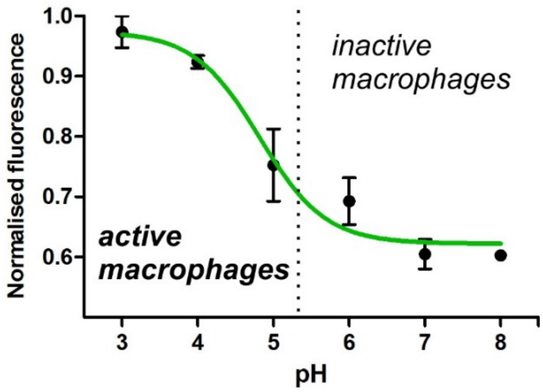

Figure S4. pH-dependent fluorescence intensity of mCCL2-MAF (**6**). Fluorescence was measured in aqueous buffers covering the pH range from 3 to 8. pK_a: 4.9±0.3. $\lambda_{exc.}$: 460 nm.

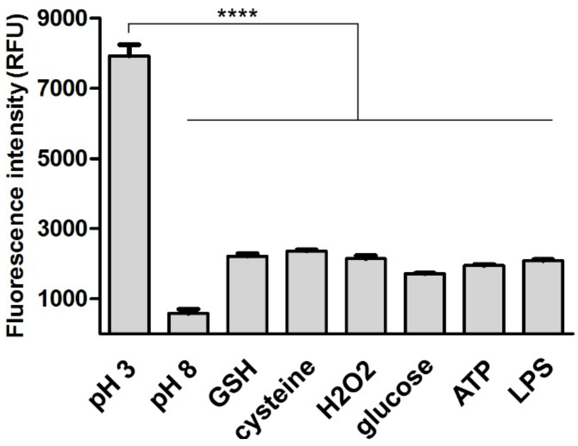

Figure S5. Fluorescence response of compound **4** (15 μM) upon incubation with different biologically relevant metabolites (all at 100 μM) and LPS (100 ng mL^{-1}). $\lambda_{exc.}$: 450 nm, $\lambda_{exc.}$: 520 nm. Values presented as means±s.e.m. (n=3). **** for $p < 0.0001$.

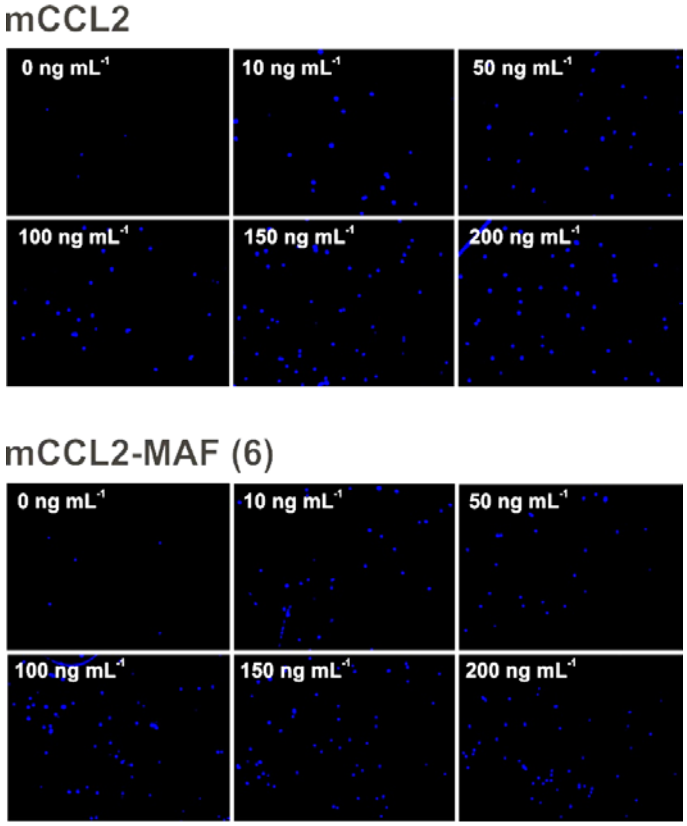

Figure S6. Representative fluorescence microscopy images from chemotactic macrophage migration assays. RAW264.7 mouse macrophages were plated in transwell plates (1.5 x 10^5 cells /well) and incubated for 2 h with mCCL2 (top panel) or mCCL2-MAF (**6**, bottom panel) at the indicated concentrations. Migrated cells were stained with DAPI and imaged under the widefield fluorescence microscope.

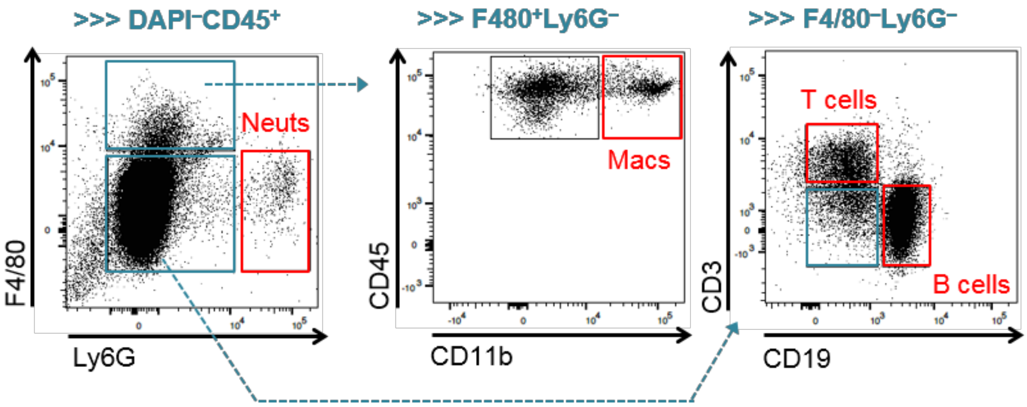

Figure S7. Flow cytometric gating strategy for the identification of immune cells in the spleen of wild-type C57/BL6 and *Ccr2* KO mice. Neuts: neutrophils, Macs: macrophages.

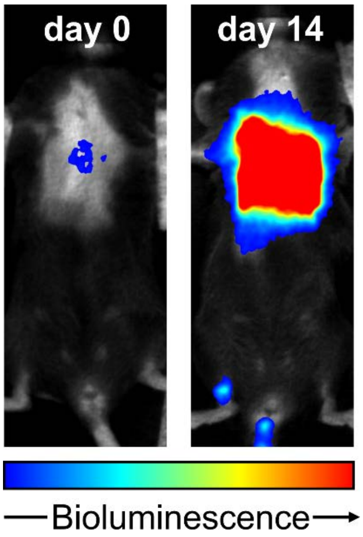

Figure S8. Representative bioluminescence images of wild-type C57/BL6 mice (from n=3) that were intravenously injected with E0771-LG mouse mammary tumour cells expressing firefly luciferase. Images at 30 min (day 0) and 2 weeks (day 14) after the injection of tumour cells are shown to indicate the formation of metastasis in the lung.

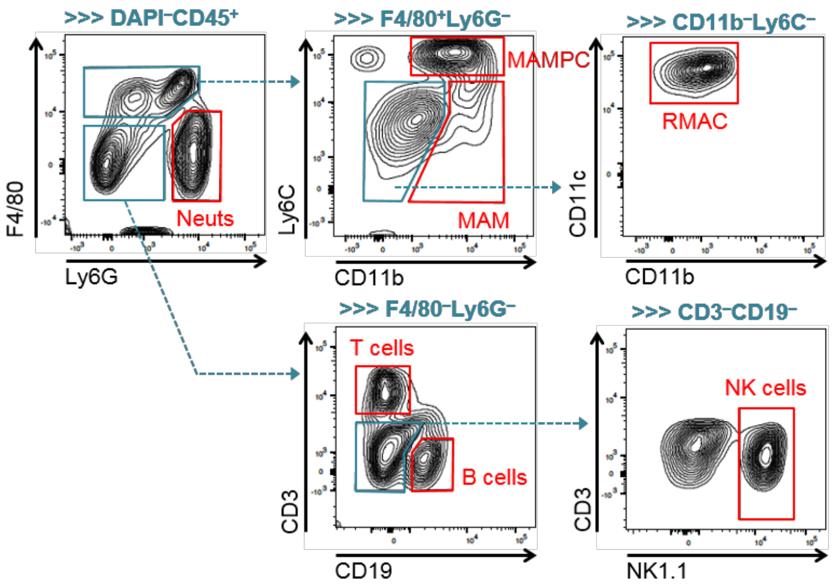

Figure S9. Flow cytometric gating strategy for the identification of immune cells in the metastatic lungs. Neuts: neutrophils, MAMPC: metastasis-associated macrophages progenitor cells, MAM: metastasis-associated macrophages, RMAC: resident macrophages NK cells: natural killer cells.

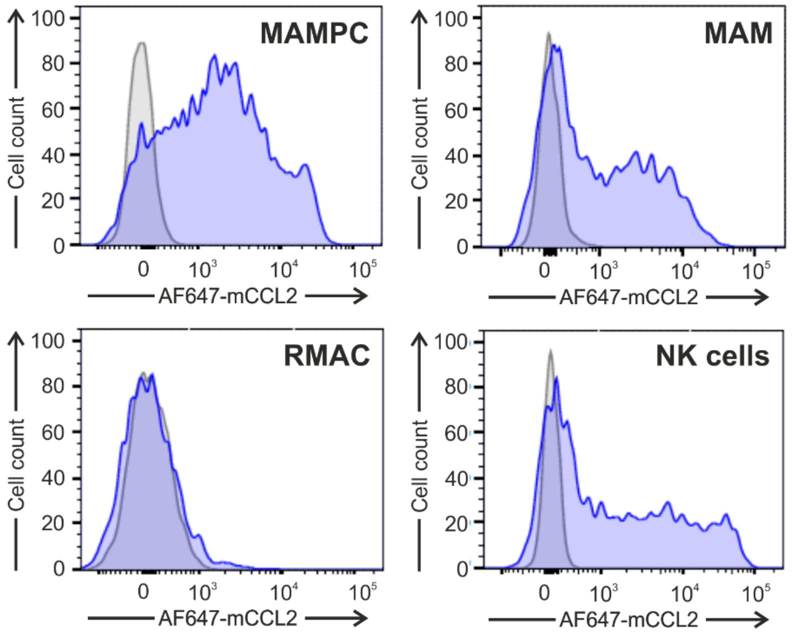

Figure S10. Representative histograms showing the fluorescence intensity of AF647-mCCL2 in different immune cells from metastatic lungs *in vivo*. Single-cell suspensions were prepared from metastatic lungs after *in vivo* tail vein injection of AF647-mCCL2. The indicated immune cell subpopulations were gated as shown in Figure S9.

Experimental Protocols for Spectroscopy and Biological Assays

Spectral characterisation. Spectroscopic yield data were recorded on a Synergy HT spectrophotometer (Biotek). Compounds were dissolved at the indicated concentrations and spectra were recorded at r.t. Spectra are represented as means from at least two independent experiments with n=3.

Cell culture of macrophages and cancer cells, and *in vitro* activation. RAW 264.7 mouse macrophages were cultured in DMEM supplemented with 10% FBS, antibiotics (100 U mL^{-1} penicillin, 100 mg mL^{-1} streptomycin) and 2 mM L-glutamine in a humidified atmosphere at 37 °C with 5% CO_2. For *in vitro* activation, macrophages were treated with LPS (100 ng mL^{-1}) for 18 h as previously reported.[5] E0771 mouse mammary adenocarcinoma cells were provided by Dr. E. Mihich (Rosewell Park Cancer Institute, NY), and their highly metastatic derivative (E0771-LG) was established through *in vitro* selections as reported.[6] In order to detect the cancer cells *in vivo*, E0771-LG cells were manipulated to express firefly luciferase and cultured in DMEM supplemented with 10% FBS.

Transwell assays. RAW264.7 mouse macrophages (1.5×10^5 cells in 100 µL of serum-free media) were plated on 24-well plates (5.0 µm membranes, Corning®). Receiver wells were filled with indicated concentrations of the chemokines and inserts were placed on. The wells were then incubated at 37°C for 2 h to allow cells migrate through the membrane. Then, inserts were removed and washed with PBS, scraped non-migrated cells with cotton buds and washed with PBS. Cells were fixed with cold acetone: MeOH (1:1) for 5 min at r.t. and washed with PBS. Finally, cells were incubated with DAPI (1:5000 dilution), washed with PBS, and membranes were cut and mounted with Mowiol® in microscope slides. Cells were counted on ImageJ after taking images on a widefield fluorescence microscope.

Fluorescence confocal microscopy. *In vitro experiments in RAW264.7 mouse macrophages.* Cells were plated on glass chamber slides Lab-Tek™ II (Nunc). When

appropriate, cells were stimulated with LPS as described above, washed with PBS and incubated with the fluorescent compounds at 37°C. Cells were imaged under a Leica SP8 fluorescence confocal microscope equipped with a live-cell imaging stage. Fluorescence and brightfield images were acquired using a 40X oil objective. Fluorophores were excited with 405 nm, 488 nm, 543 nm, or 633 nm lasers. Images were analysed and processed with ImageJ.

Ex vivo staining of metastatic lungs. Metastatic lungs were harvested from mice, mounted in OCT and immediately frozen on dry ice. After cryosection (5 μm thickness), tissues were fixed in ice-cold paraformaldehyde (4% in PBS) for 15 min and dried. PFA was removed in xylene (×2), followed by different solutions of EtOH [100% (x2), 95%, 80% and 75%] and finally in PBS. Slides were blocked in 5% (v/v) donkey serum 0.1% Tween-20 in PBS for 1 h at r.t. prior to the addition of antibodies [e.g., rat anti-mouse F4/80 antibody (1:100)], which were incubated overnight at 4°C. Slides were washed in PBS (3 × 5 min) before addition of the secondary antibody (e.g., anti-rat Alexa Fluor 647 secondary antibody), which was incubated for 1 h at r.t. Slides were washed in PBS (3 × 5 min), and tissue autofluorescence was quenched using the Vector® TrueVIEW™ Autofluorescence Quenching Kit (1:4 dilution in PBS) for 3 min. Slides were washed in PBS and mounted in VECTASHIELD® Vibrance™ Antifade Mounting Medium. Fluorescence images were acquired on a Leica SP8 confocal microscope and analysed with Image J.

Flow cytometry. *In vitro experiments in RAW264.7 mouse macrophages.* Unstimulated and activated RAW264.7 macrophages were incubated with the probes at the indicated concentrations for 30 min at 37°C alongside appropriate controls. Bafilomycin A1 (100 nM) and rat anti-mCCR2 (1:40 dilution, BioLegend) were used to block phagosomal acidification and receptor-mediated endocytosis, respectively. Samples were spun at 300 g for 5 min, and the pellets were resuspended in FACS buffer (1% BSA) prior to flow cytometry. Flow cytometry was performed in a 5 LSR Fortessa and analysed using Flowjo software.

Ex vivo staining of mouse spleens. Spleens from C57BL/6 or *Ccr2* KO mice (16-week-old female) were smashed through 70 μm filters. After removing red blood cells in the single-cell suspensions by RBC lysis buffer, 5×10^5 cells were incubated in 50 μL of α-MEM including 10% FBS with 100 nM of mCCL2-MAF (**6**) or AF647-CCL2 at 37°C for 1 h. Cells were then washed by staining buffer (PBS including 2% bovine serum albumin) and re-suspended in 100 μL of the same buffer. These cells were incubated with anti-mouse CD16/CD32 antibody (BD Bioscience) on ice for 30 min to block non-specific antibody binding and incubated for 30 min with the indicated fluorescent antibodies as markers of immune cells. Cells were washed and re-suspended in FACS buffer including DAPI. To identify specific immune cell populations, we used antibodies (Biolegend) for the following antigens: CD45 (30-F11), F4/80 (BM8), CD11b (M1/70), Ly6C (HK1.4), Ly6G (1A8), CD11c (N418), CD3 (17A2), CD19 (6D5), NK1.1 (PK136). Flow cytometry was performed in a LSRII cytometer and analysed using Flowjo software.

In vivo staining of mouse metastatic lungs. C57BL/6 mice with lung metastatic tumours (day 14 after injection of tumour cells) were intravenously injected with 10 μg of mCCL2-MAF (**6**) or AF647-CCL2. After 2 h, lungs were isolated, digested by enzyme (Lung Dissociation Kit, Miltenyi), and filtrated with a 40 μm cell strainer. After removing red blood cells, 5×10^5 cells were incubated with anti-mouse CD16/CD32 antibody and fluorescent antibodies as the immune cell markers as described above. Flow cytometry was performed in a LSRII cytometer and analysed using Flowjo software.

Supplementary References

1. A. Vázquez-Romero, N. Kielland, M.J. Arévalo, S. Preciado, R.J. Mellanby, Y. Feng, R. Lavilla, M. Vendrell, *J. Am. Chem. Soc*. **2013**, *135*, 16018−16021.

2. L.I. Johnson-Cadwell, M.B. Jekabsons, A. Wang, B.M. Polster, D.G. Nicholls, *J. Neurochem.* **2007**, *101*, 1619-1631.

3. M.L. Denton, K.J. Schuster, B.A. Rockwell, *J. Microsc.* **2006**, *221*, 164-171.

4. S.V. Kalivendi, S, Kotamraju, S. Cunningham, T. Shang, C.J. Hillard, B. Kalyanaraman, *Biochem. J.* **2003**, *371*, 151-164.

5. Y. Chen, W. Liu, Y. Wang, L. Zhang, J. Wei, X. Zhang, F. He, L Zhang, *Cell. Signal.* **2017**, *33*, 107-121.

6. T. Kitamura, B.Z. Qian, D. Soong, L. Cassetta, R. Noy, G. Sugano, Y. Kato, J. Li, J.W. Pollard, *J. Exp. Med.* **2015**, *212*, 1043-1059.
